# Cationic Catalysts as a New Strategy to Catalyze *N*-Carboxyanhydrides Polymerization

**DOI:** 10.1021/acscentsci.4c01926

**Published:** 2024-12-09

**Authors:** Haohui Ye, Jianfeng Cai

**Affiliations:** Department of Chemistry, University of South Florida, Tampa, Florida 33620, United States

Synthetic polypeptides, designed to mimic the function of natural polypeptides and proteins, play a pivotal role in advancing biotechnology, enabling more innovative solutions for research and application in biomedical challenges.^1,2^ As one major route to synthetic polypeptides, primary amines-initiated *N*-carboxyanhydrides (NCA) polymerization has the advantage of fabricating polypeptides with multiple designable structures, offering potential strategies for designing polypeptides with flexible structures.^3^

However, several challenges persist in the synthesis process, including the difficulty of obtaining high molecular weight polypeptides due to their sensitivity to moisture and slow reaction rates, as well as side reactions caused by the basicity of primary amines, which can deprotonate NCAs. Inducing catalysts is one of the most useful and common methods to accelerate reaction rates. Guanidine, a strong base and hydrogen bonding acceptor, is insensitive to air and has been used for catalyzing the polymerization of cyclic esters. 1,1,3,3-Tetramethyl guanidine has been induced to catalyze polymerization of NCA monomers because it can increase the initiation and propagation rate, which are two key stages to ring-opening polymerization (ROP).^4^

Furthermore, the controllability of polymerization can also be guaranteed by catalysts consisting of two synergetic compounds. The rate of polymerization can be controlled by an adjustable initiator system between the primary ammonium salt and tertiary amine. With different ratios of primary amines in combination with its corresponding ammonium salt, controlled polypeptides can be polymerized with designated end groups and predetermined molecular weights. For catalyzing NCA polymerization, the interaction between catalysts and NCAs or initiators plays a major role, mostly in hydrogen bonding.^5^ However, hydrogen bonding also exists between adjacent catalysts or within catalysts themselves, usually affecting their catalytic performance.

Although many efforts have been devoted to NCA polymerization, concurrently achieving fast and precisely controlled NCA polymerization, initiated by primary amines, for the synthesis of high molecular weight polypeptides is still an obvious obstacle. Therefore, it is necessary to seek new catalytic strategies for high-efficiency NCA polymerization with primary amines initiation, to achieve the synthesis of higher molecular weight polypeptides with fewer side effects.

**Figure 1:**
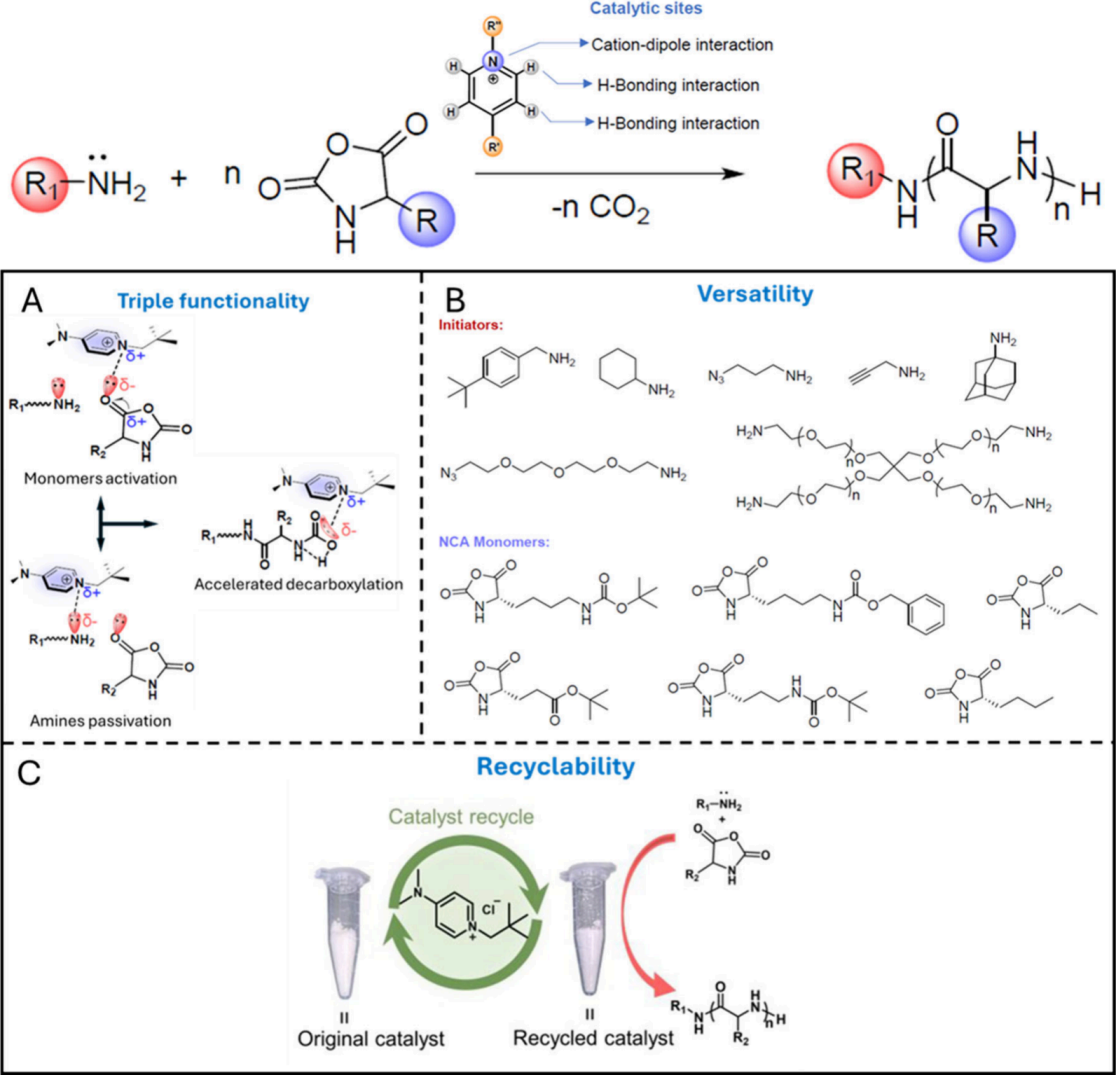
Primary amine initiated NCA ROP polymerization catalyzed by a single-center trifunctional organocatalyst. The organocatalyst demonstrates A) triple functionality, B) versatility with different combinations of initiators and monomers, C) recyclability. Revised with permission from ref (1). Copyright 2024 American Chemical Society.

Facing these obstacles, Liu, Wu, and co-workers developed a new class of catalysts which use cation-dipole interactions to overcome long-standing challenges in primary amine-initiated NCA polymerization, as detailed in the paper “Single-Center Trifunctional Organocatalyst Enables Fast and Controlled Polymerization on *N-*Carboxyanhydride”, published in *ACS Central Science*.^1^ The authors focused on achieving fast and controlled NCA polymerization with the help of a conjugated cationic catalyst (Figure 1). Not only did the cation-dipole interaction enhance the NCA polymerization rate, but also a predictable molecular weight could be achieved along with narrow dispersity, showing the remarkable controllability of NCA polymerization. The single center catalysts with their triple functions fascinated and enhanced NCA polymerization by activating the monomer’s electrophilicity, accelerating the decarboxylation of intermediates, and improving polymerization controllability by diminishing side reactions.

The triple functions of this class of catalyst were explored, starting with the controllable NCA polymerization of γ-benzyl-l-glutamate *N*-carboxyanhydride (BLG) with different catalysts. Among all the selected cationic catalysts, 4-dimethylamino-1-neopentylpyridinium chloride (DMAPPCl) showed a dramatic improvement in the polymerization efficacy. With a controlled [M]:[I] feed ratio and the presence of DMAPPCl, a series of polypeptides were obtained with linearly increasing molecular weight, showing the cationic catalyst’s contribution to the high controllability. The versatility of DMAPPCl catalyzation in NCA polymerization was verified by selecting various primary amine initiators. With the [M]:[I] feed ratio varying from 20:1 to 500:1, poly-γ-benzyl-l-glutamates (PBLGs) were produced with controllable molecular weights from 4.5 to 109.3 kg/mol. Besides the excellent controllability of DMAPPCl-catalyzed NCA polymerization, its product also showed well-defined secondary structures, which was demonstrated by BLG NCA with varied chain lengths (DP as 21, 54, and 101) showing α-helix structures.

The versatility of the DMAPPCl catalyst to NCA polymerization was investigated by various primary amine initiators (Figure 1B) with a controlled feed ratio of [M]:[I] = 50:1. Even with different initiators, even some with higher steric hindrance, the polymerization could be completed within 40 min and produced polypeptides with designated molecular weights and narrow dispersity. It is worth noting that DMAPPCl-catalyzed polymerization could be achieved with various solvents possessing different polarities (especially in weak polar solvents), even with undried solvents. Notably, even after recycling five times by simple centrifugation after polymerization, the recycled DMAPPCl (Figure 1C) maintained a robust catalytic performance, and the obtained polypeptides retained controlled molecular weights and narrow dispersity.

The catalytic mechanism was studied through low temperature ^13^C NMR spectra analysis and DFT calculations. As shown in Figure 1A, DMAPPCl can both activate NCAs and moderately passivate primary amines in the initiation stage, with rapid dynamic transfer between these two states. It also assists in the intramolecular transfer of hydrogen atoms and reduces the energy barrier of decarboxylation, thereby accelerating CO_2_ release. The energy barrier of the chain propagation stage was also studied, and it was found that the energy barrier of nucleophilic addition to NCAs and intramolecular decarboxylation were decreased by 11.9 and 7.4 kcal/mol, respectively.

Overall, this work presents a new class of multifunctional catalysts with only one single center, which could achieve a convenient route to highly efficient and well-controlled NCA polymerization for synthetic polypeptides. By activating NCAs and moderately passivating primary amines together, along with the accelerated decarboxylation, the polymerization rate was remarkably improved. This advancement not only addresses current challenges in NCA polymerization but also broadens the scope of polymerization chemistry, paving the way for the development of novel polymeric materials with enhanced functionalities.
